# Supplementary material for: Mitochondrial dysfunction underlying sporadic inclusion body myositis is ameliorated by the mitochondrial homing drug MA-5
Source: PLoS One. 2020 Dec 2;15(12):e0231064. doi: 10.1371/journal.pone.0231064 (PMC7710105; doi:10.1371/journal.pone.0231064)
Supplement: S1 File — (PDF) [file pone.0231064.s004.pdf]

Supplementary Data sets

Title: Sporadic inclusion body myositis underling mitochondrial dysfunction ameliorated by mitochondrial homing drug, MA-5

Yoshitsugu Oikawa[1,2], Rumiko Izumi[3], Masashi Koide[4], Yoshihiro Hagiwara[4], Makoto Kanzaki[5], Naoki Suzuki[3], Koichi Kikuchi[2], Tetsuro Matsuhashi[1], Yukako Akiyama[2], Mariko Ichijo[2], Takafumi Toyohara[2,6], Takehiro Suzuki[2,6],
Eikan Mishima[2], Yasutoshi Akiyama[2], Yoshiaki Ogata[6,7],
Chitose Suzuki[2], Hironori Hayashi[8,9], Eiichi N. Kodama[8],
Ken-ichiro Hayashi[10], Eiji Itoi[4], Masashi Aoki[3], Shigeo Kure[1] &
Takaaki Abe*[2,6,7]

[1]Department of Pediatrics, Tohoku University Graduate School of Medicine, Sendai, 980-8574, Japan
[2]Division of Nephrology, Endocrinology and Vascular Medicine, Tohoku University Graduate School of Medicine, Sendai 980-8574, Japan
[3]Division of Neurology, Tohoku University Graduate School of Medicine, Sendai, 980-8574, Japan
[4]Department of Orthopedics, Tohoku University Graduate School of Medicine, Sendai, 980-8574, Japan
[5]Department of Biomedical Engineering, Tohoku University Graduate School of Biomedical Engineering, Sendai, 980-8574, Japan
[6]Department of Medical Science, Tohoku University Graduate School of Biomedical Engineering, Sendai, 980-8574, Japan
[7]Department of Clinical Biology and Hormonal Regulation, Tohoku University Graduate School of Medicine, Sendai 980-8574, Japan
[8]Division of Infectious Diseases, International Institute of Disaster Science, Graduate School of Medicine, Tohoku Medical Megabank Organization, Tohoku University, Sendai 980-8574, Japan
[9]Department of Intelligent Network for Infection Control, Graduate School of Medicine, Tohoku University, Sendai 980-8574, Japan
[10]Department of Biochemistry, Okayama University of Science, Okayama 700-0005, Japan

Supplementary Figure 1

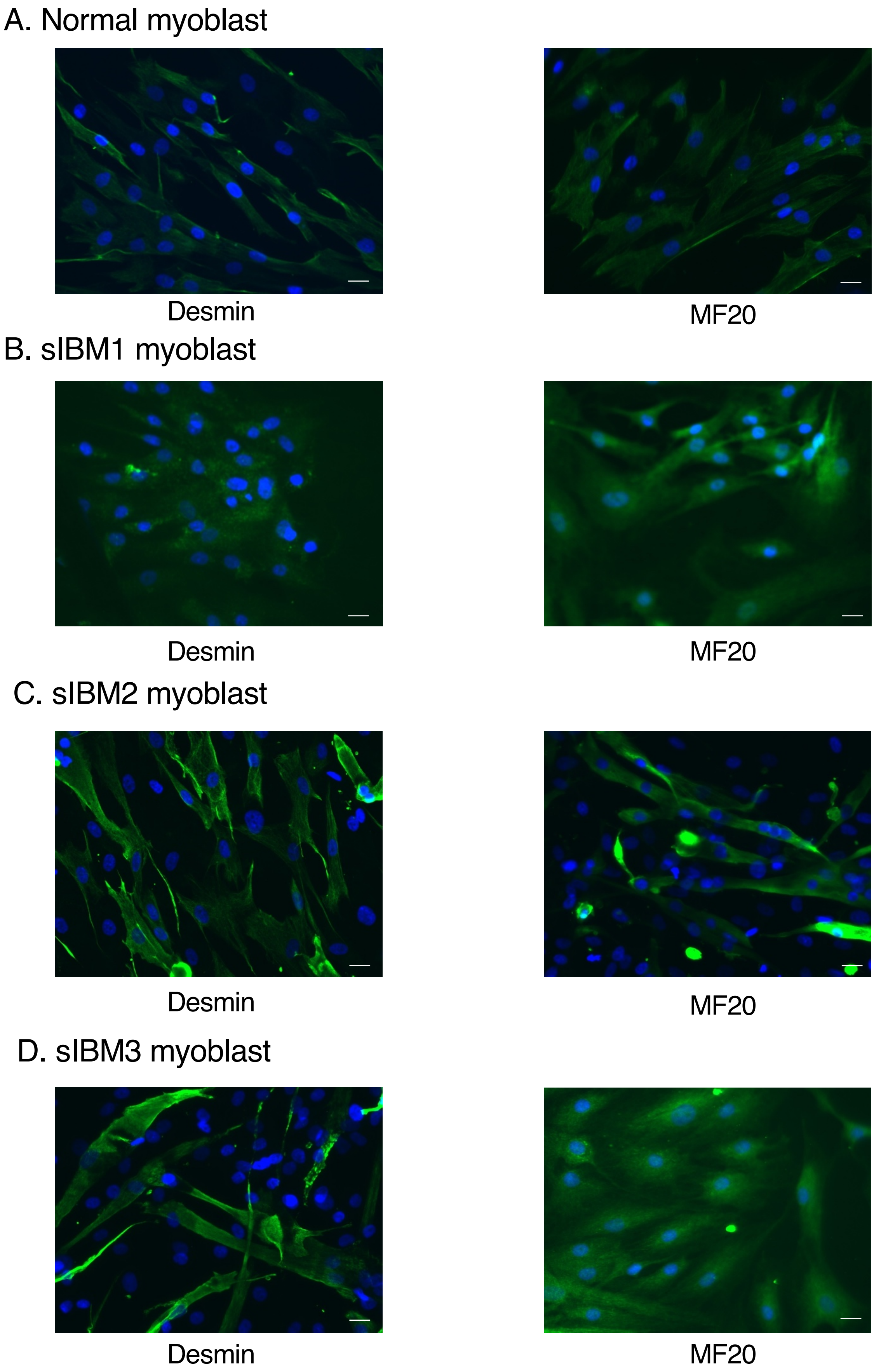

Figure S1

Immunostaining in sIBM patient’s myoblasts.
Scale bars represent 50 μm.

Supplementary Figure 2

Case2

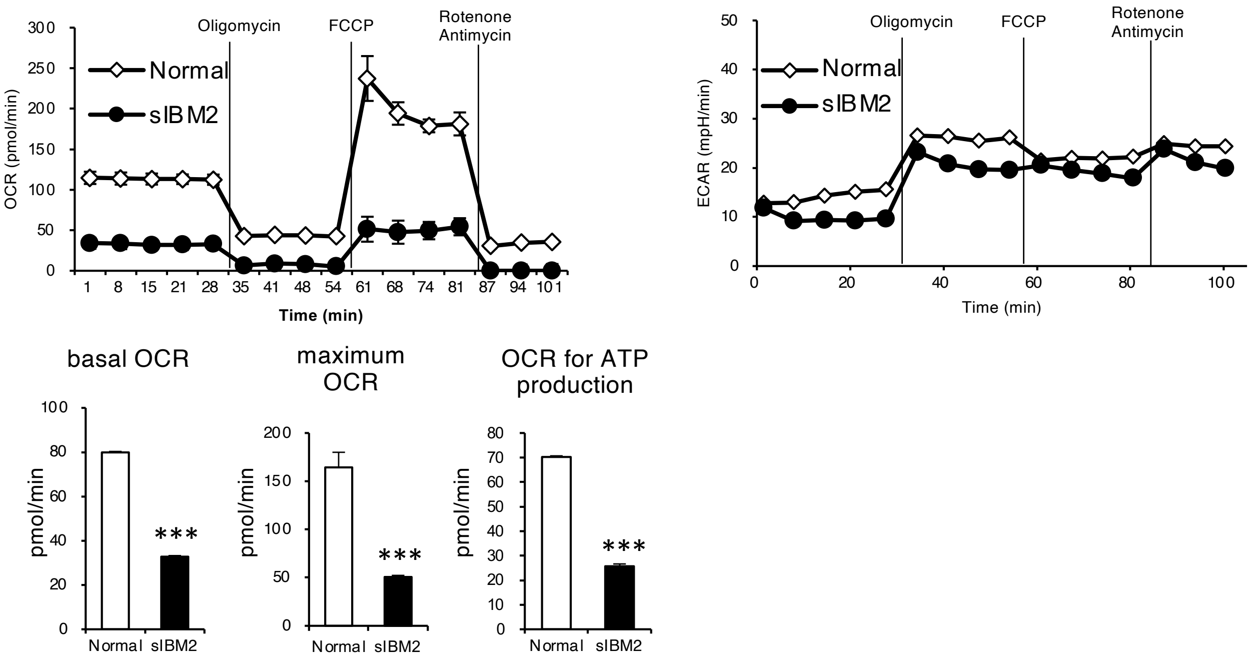

Case3

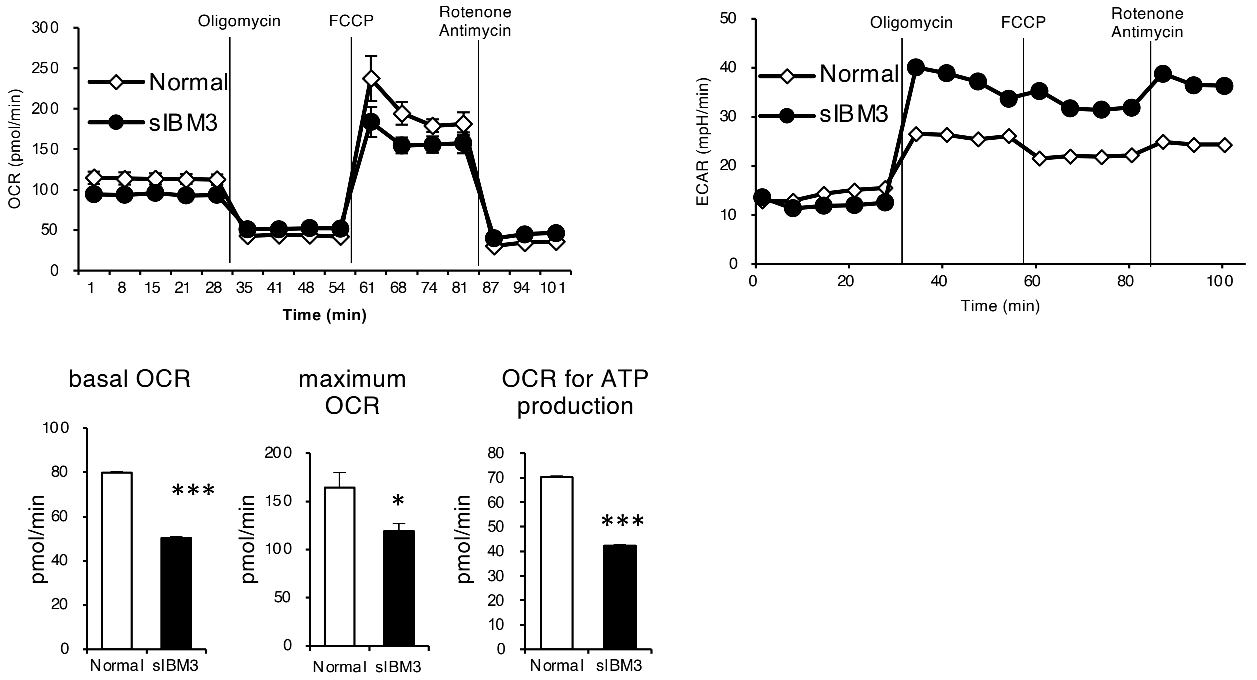

Figure S2

sIBM myoblasts on mitochondrial respiration.

Bioenergetic assay with normal and sIBM patient myoblasts (sIBM2, sIBM3) in comparison with OCR (left) and ECAR (right). The data represent the mean ± SEM. *$p < 0.05$, ***$p < 0.001$ (unpaired two-tailed Student's *t*-test versus Normal).

Supplementary Figure 3

Case 2

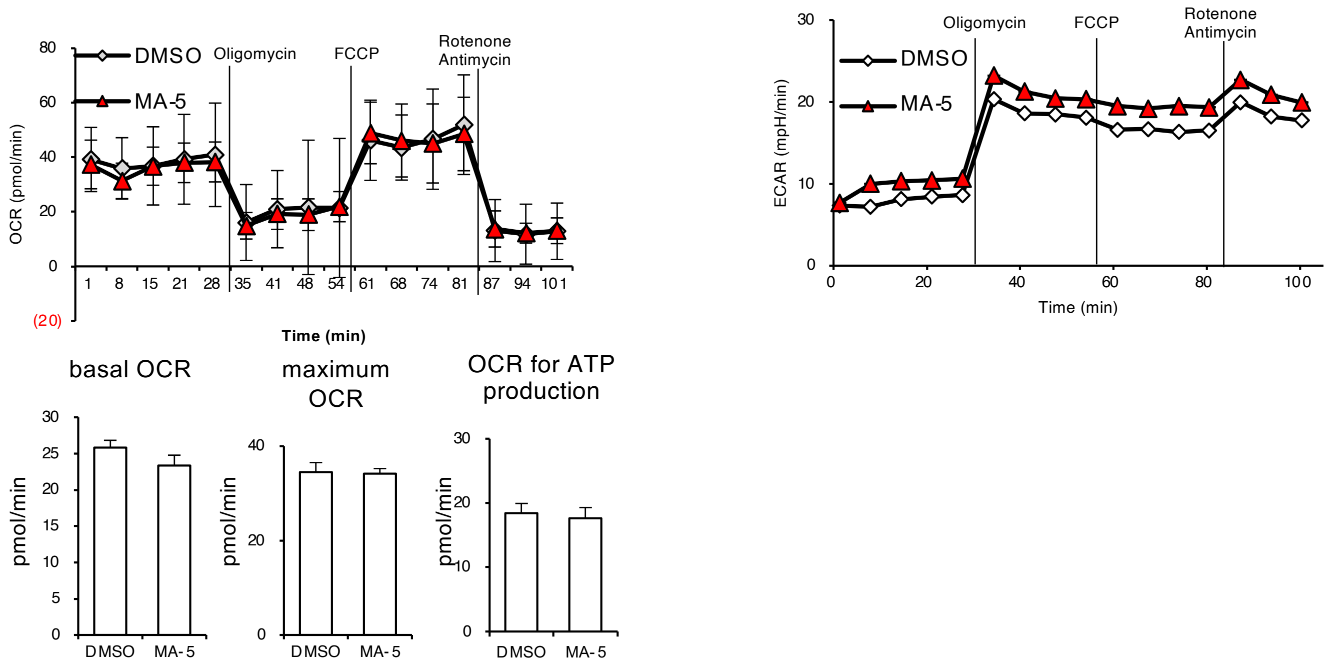

Case 3

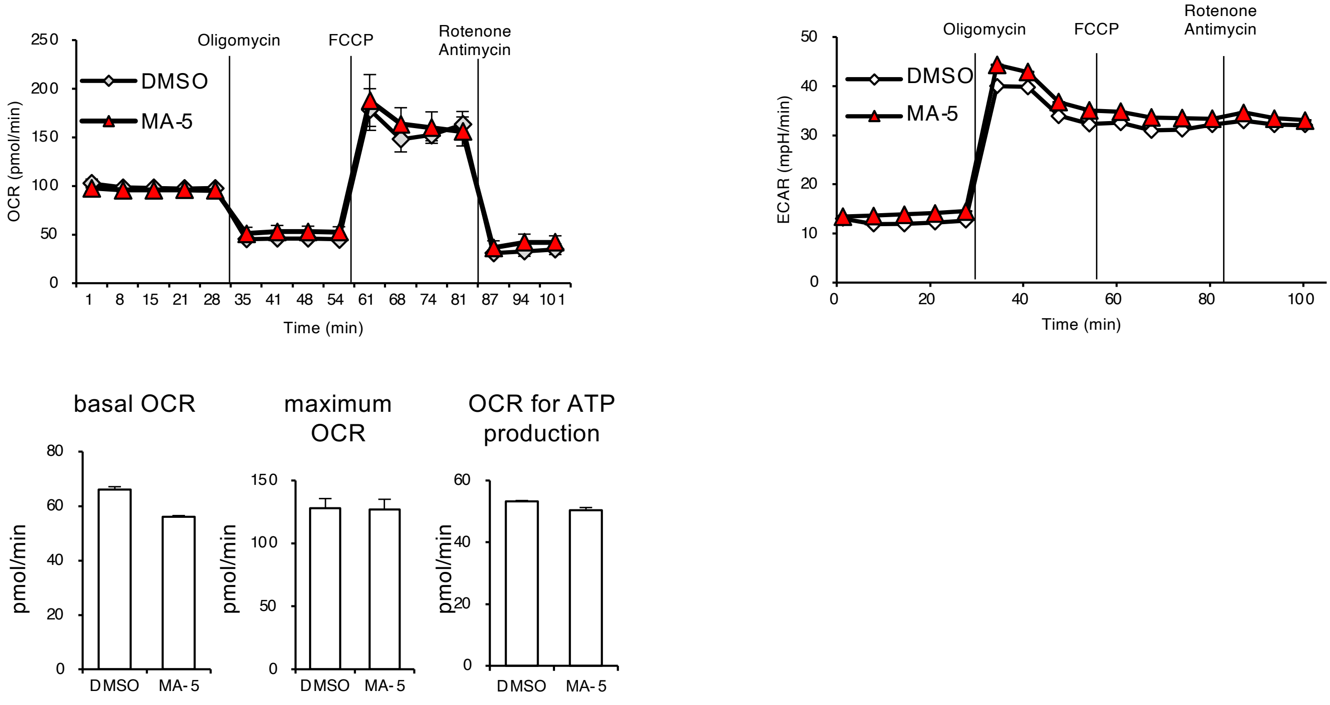

Figure S3
Effect of MA-5 on mitochondrial respiration.
Bioenergetic assay with myoblasts from sIBM patient (case2, case3) treatment DMSO and MA-5 in comparison with OCR (left) and ECAR (right). The data represent the mean ± SD.

Supplementary Figure 4

Case 2

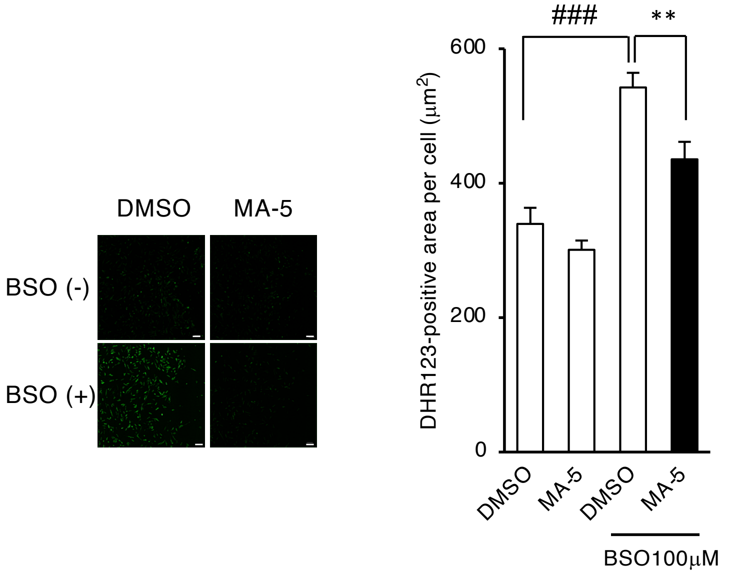

Case 3

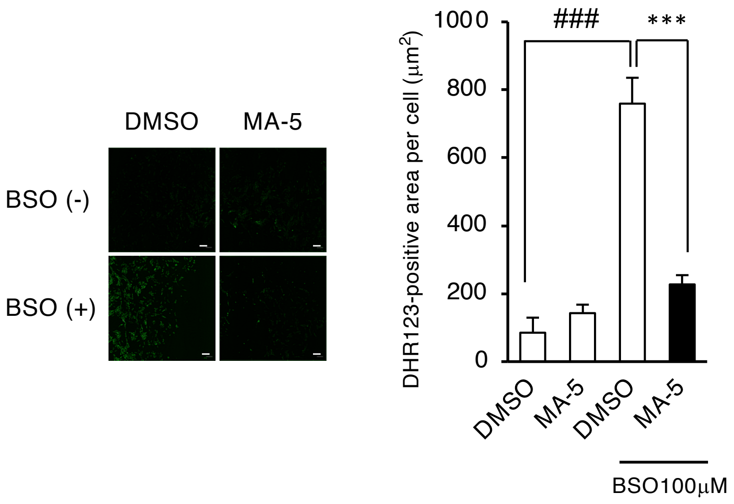

Figure S4

Effect of MA-5 on mtROS.

MA-5 improved ROS production by BSO. DHR123 staining of sIBM myoblasts with DMSO (upper left), MA-5 (upper right), BSO + DMSO (lower left), BSO + MA-5 (lower right). The data represent the mean ± SEM. ###p < 0.001 (unpaired two-tailed Student's t-test versus DMSO), **p < 0.01, ***p < 0.001 (unpaired two-tailed Student's t-test versus BSO + DMSO). Scale bars represent 200 μm.

Supplementary Figure 5

A. 0.1% DMSO

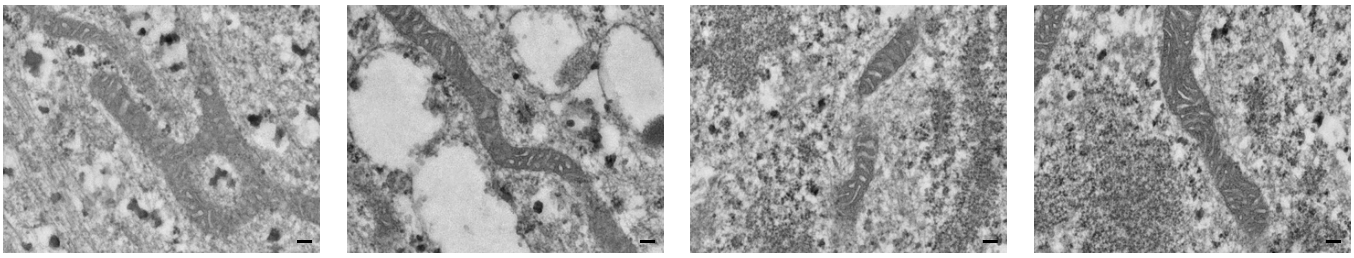

B. MA-5 10μM

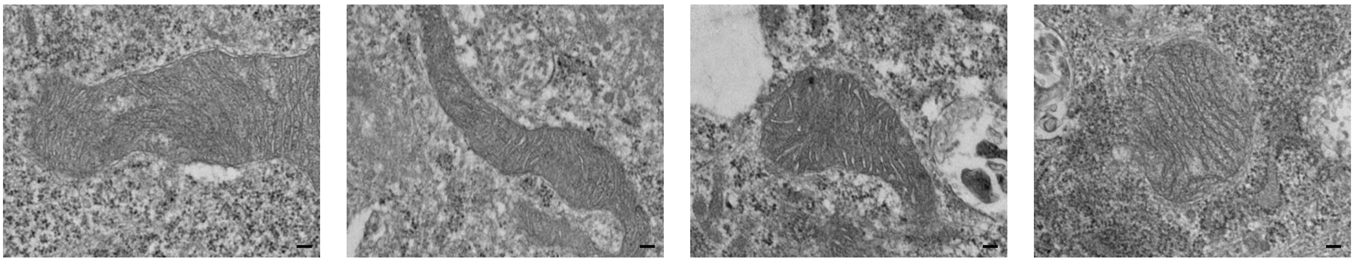

Figure S5
Electron microscopy of sIBM myoblasts.
Structural analysis of cristae of sIBM myoblasts by electroscopic analysis.
The sIBM myoblasts were treated with (A) 0.1% DMSO (B) 10 μM MA-5 for 24 h.
The comparison with maximal width and length of cristae measured by ImageJ software (n=100). Scale bars represent 500 nm

Supplementary Figure 6

A

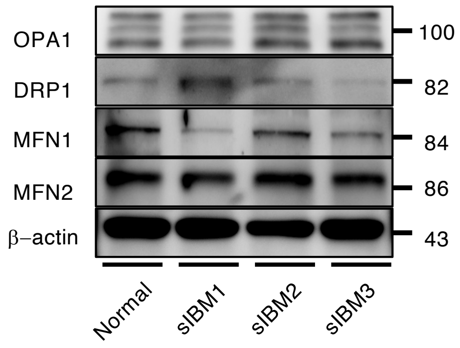

B. sIBM1 myoblast

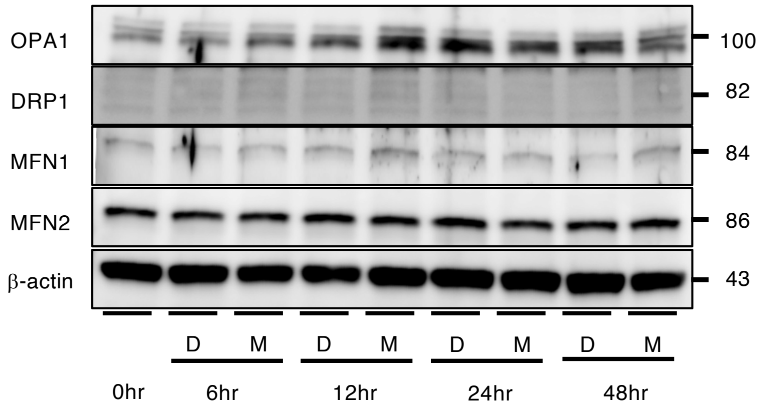

C. sIBM2 myoblast

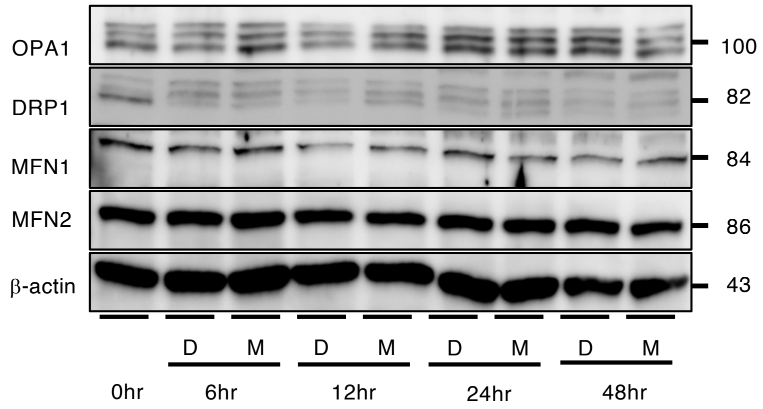

D. sIBM3 myoblast

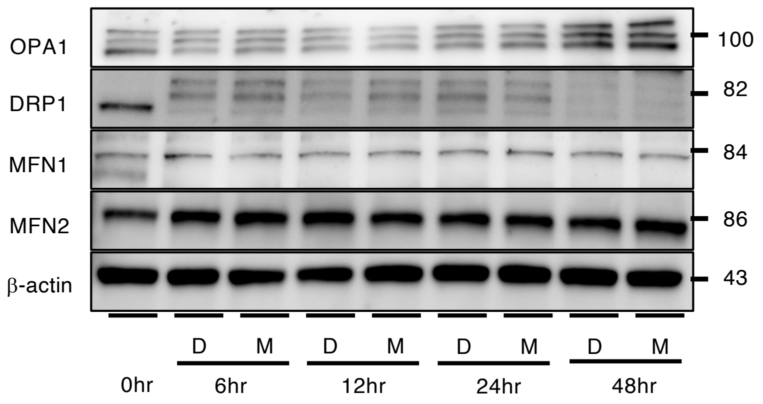

Figure S6
Effect of MA-5 on Mitochondrial related protein
A. Western blotting of individual myoblasts from normal control and sIBM patients for OPA1, DRP1, MFN1, MFN2. β-actin was used as a loading control.
B. Protein expression change in sIBM1
C. Protein expression change in sIBM2.
D. Protein expression change in sIBM3

Supplementary Figure 7

A. sIBM1 myoblast

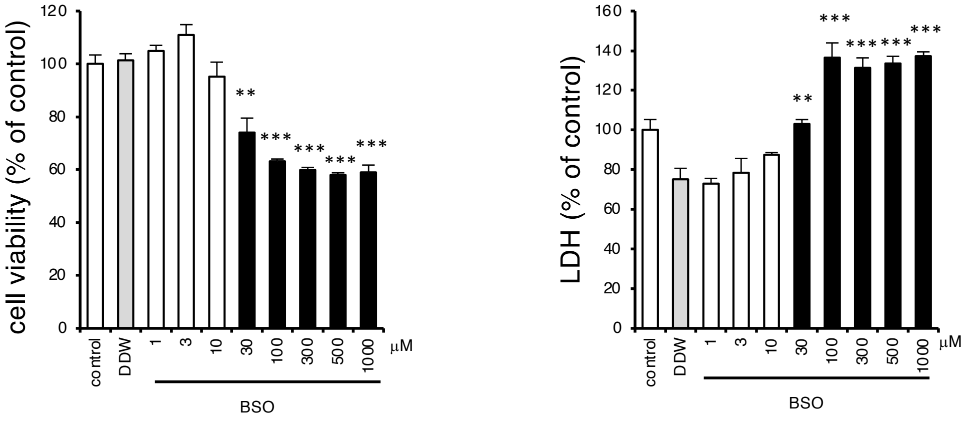

B. sIBM2 myoblast

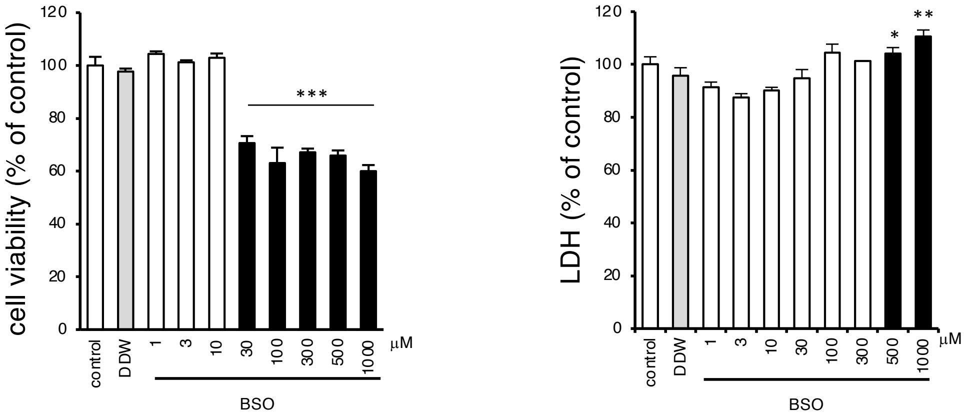

C. sIBM3 myoblast

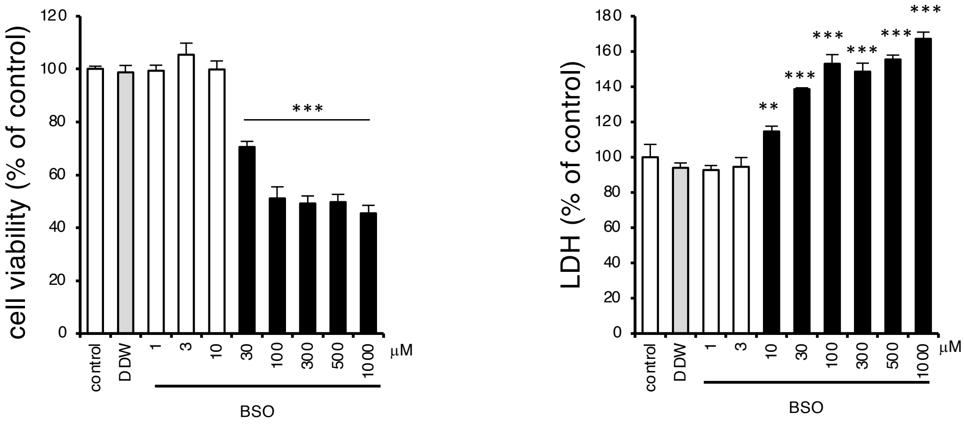

Figure S7

Cell death by BSO-treatment.

The figure on the left is the cell viability assay and on the right is the level of LDH in culture medium under oxidative stress induced by BSO-treatment. The data represent the mean $\pm$ SEM. *$p < 0.05$, **$p < 0.01$ and ***$p < 0.001$ (unpaired two-tailed Student's *t*-test versus DDW, n=4). The black square indicates significantly increased or decreased compared with DDW.

Supplementary Figure 8

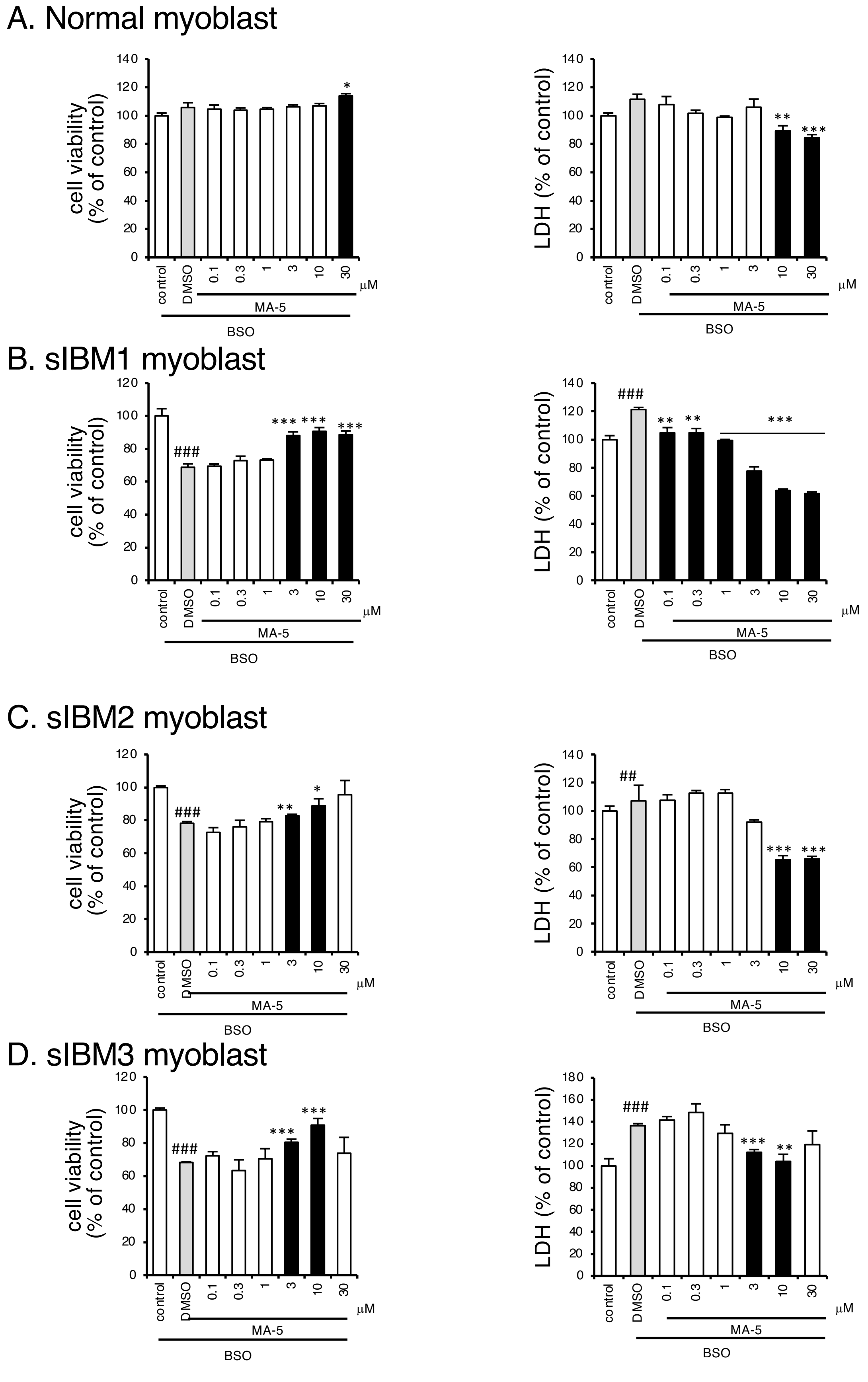

Figure S8

Cell-protective of MA-5 in sIBM myoblasts. The figure on the left is the cell viability assay and on the right is the level of LDH in culture medium under oxidative stress induced by BSO-treatment. The BSO concentration required to reach approximately 50-80 % cell death varied with the myoblasts type. The data represent the mean ± SEM. ##$p < 0.01$ and ###$p < 0.001$ (unpaired two-tailed Student's *t*-test versus control; n=4). *$p < 0.05$, **$p < 0.01$ and *** $p < 0.001$ (unpaired two-tailed Student's *t*-test versus BSO+DMSO, n=4). The black square indicates significantly increased or decreased compared with DMSO.

Supplementary Figure 9

A. 0.1% DMSO

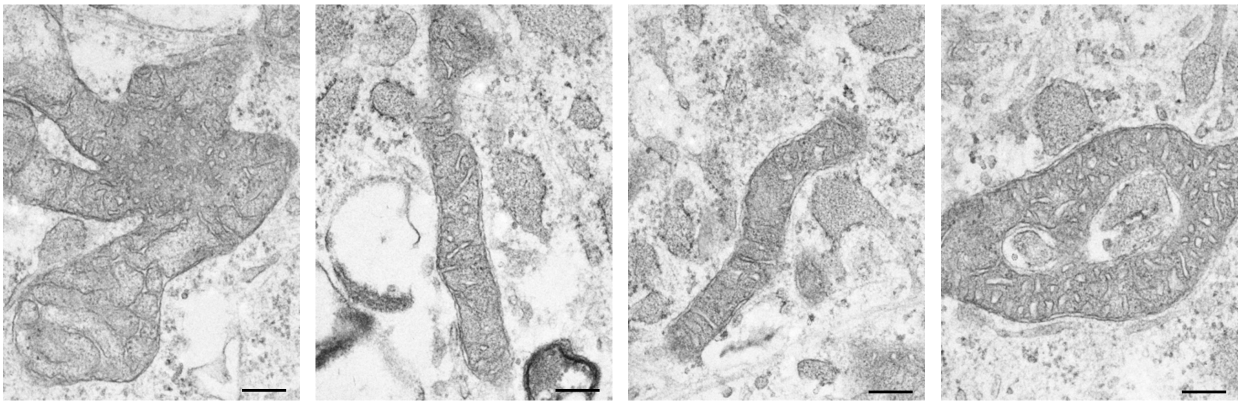

B. MA-5 10μM

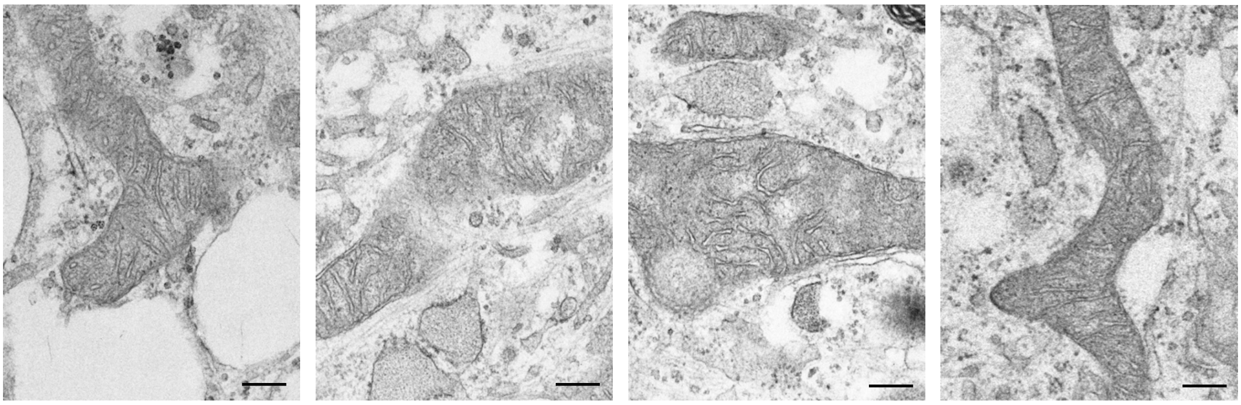

Figure S9
Electron microscopy of sIBM fibroblasts.
Structural analysis of cristae of sIBM fibroblasts by electroscopic analysis.
The sIBM fibroblasts were treated with (a) 0.1% DMSO (b) 10 μM MA-5 for 24 h.
The comparison with maximal width and length of cristae measured by ImageJ software (n=100). Scale bars represent 200 nm

Table S1

case	cell ID	pathogenic	non pathogenic
1	sIBM1	-	m.1438A>G
2	sIBM2	-	m.1382A>C<br>m.1438A>G<br>m.3849G>A
3	sIBM3	-	m.1438A>G

Table S2

A. cDNA taqman primer

Gene (Human)	
OPA1	Hs01047018_m1
MFN2	Hs00208382_m1
GAPDH	Hs02758991_g1

B. cDNA SYBR green primer

Primer (Human)	Source
DRP1 Forward: TCAACCTCCGTCTACTC	Nihon Gene Research Laboratories
DRP1 Reverse: GATCTGGAACTCGATGTCGGG	Nihon Gene Research Laboratories

Table S3

A. PCR primers for mitochondrial analysis by NGS

MTL-F1	5’-AAAGCACATACCAAGGCCAC-3’
MTL-F2	5’-TATCCGCCATCCCATACATT-3’
MTL-R1	5’-TTGGCTCTCCTTGCAAAGTT-3’
MTL-R2	5’-AATGTTGAGCCGTAGATGCC-3’
