# Supplementary figures and images for: Mitochondrial dysfunction underlying sporadic inclusion body myositis is ameliorated by the mitochondrial homing drug MA-5
Source: PLoS One. 2020 Dec 2;15(12):e0231064. doi: 10.1371/journal.pone.0231064 (PMC7710105; doi:10.1371/journal.pone.0231064)

## Slide 1
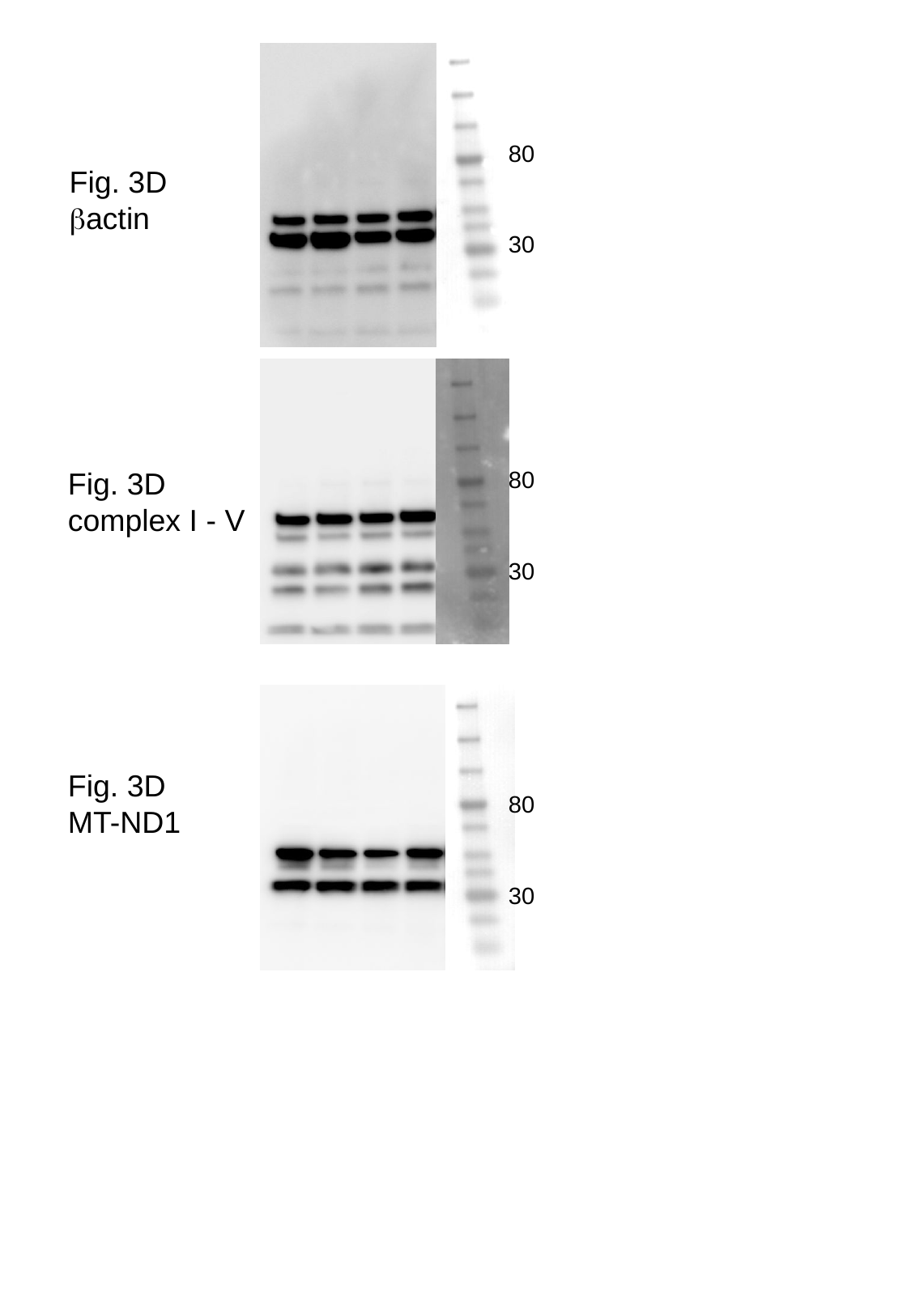

80
Fig. 3D
bactin
30
Fig. 3D
complex I - V
80
30
Fig. 3D
MT-ND1
80
30

Supplement: S2 File — (PPTX) [file pone.0231064.s005.pptx]
