# Supplementary material for: Mitochondrial dysfunction underlying sporadic inclusion body myositis is ameliorated by the mitochondrial homing drug MA-5
Source: PLoS One. 2020 Dec 2;15(12):e0231064. doi: 10.1371/journal.pone.0231064 (PMC7710105; doi:10.1371/journal.pone.0231064)
Supplement: S3 File — (PPTX) [file pone.0231064.s006.pptx]

## Slide 1
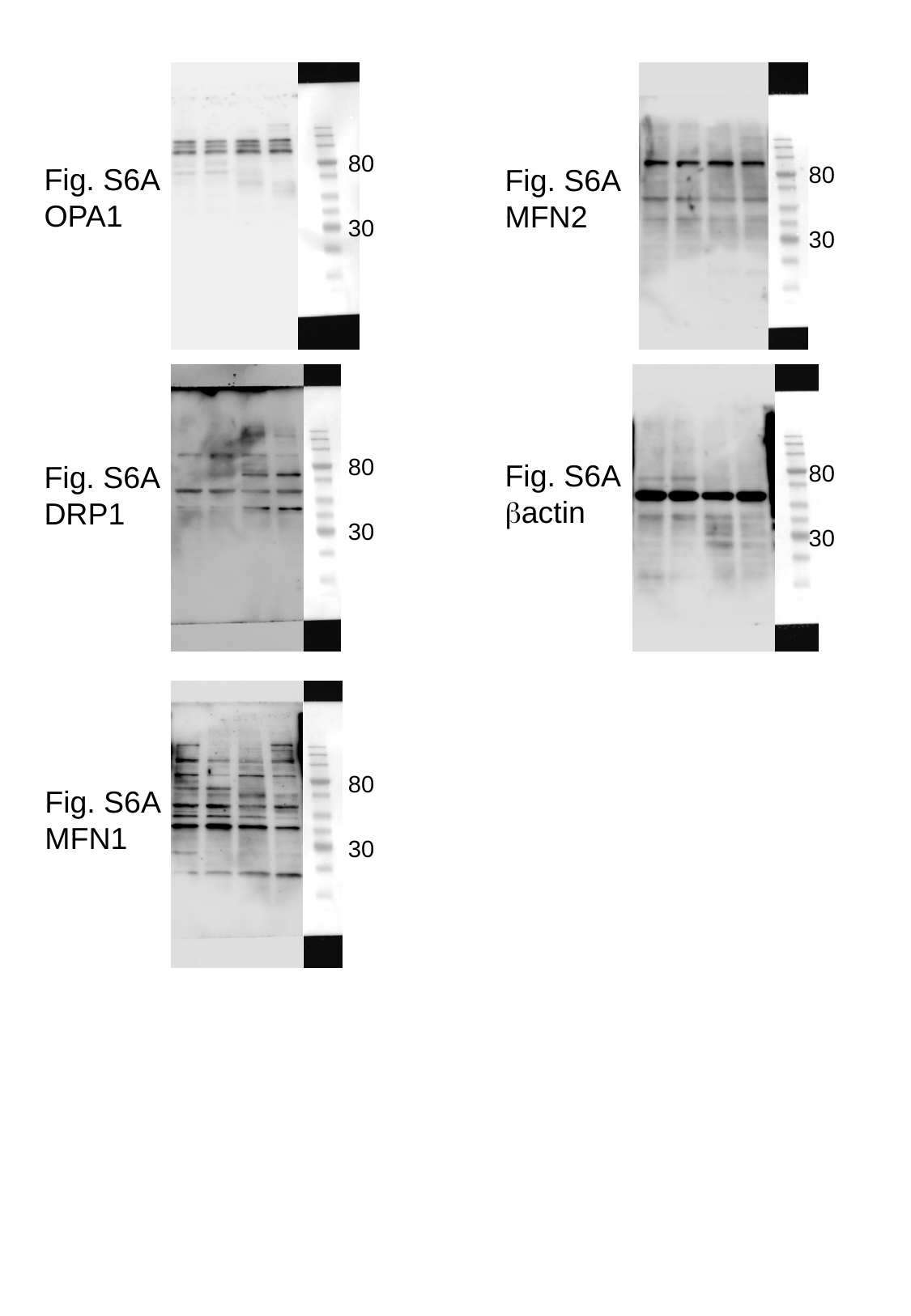

80
Fig. S6A
OPA1
80
Fig. S6A
MFN2
30
30
80
Fig. S6A
bactin
Fig. S6A
DRP1
80
30
30
80
Fig. S6A
MFN1
30

## Slide 2
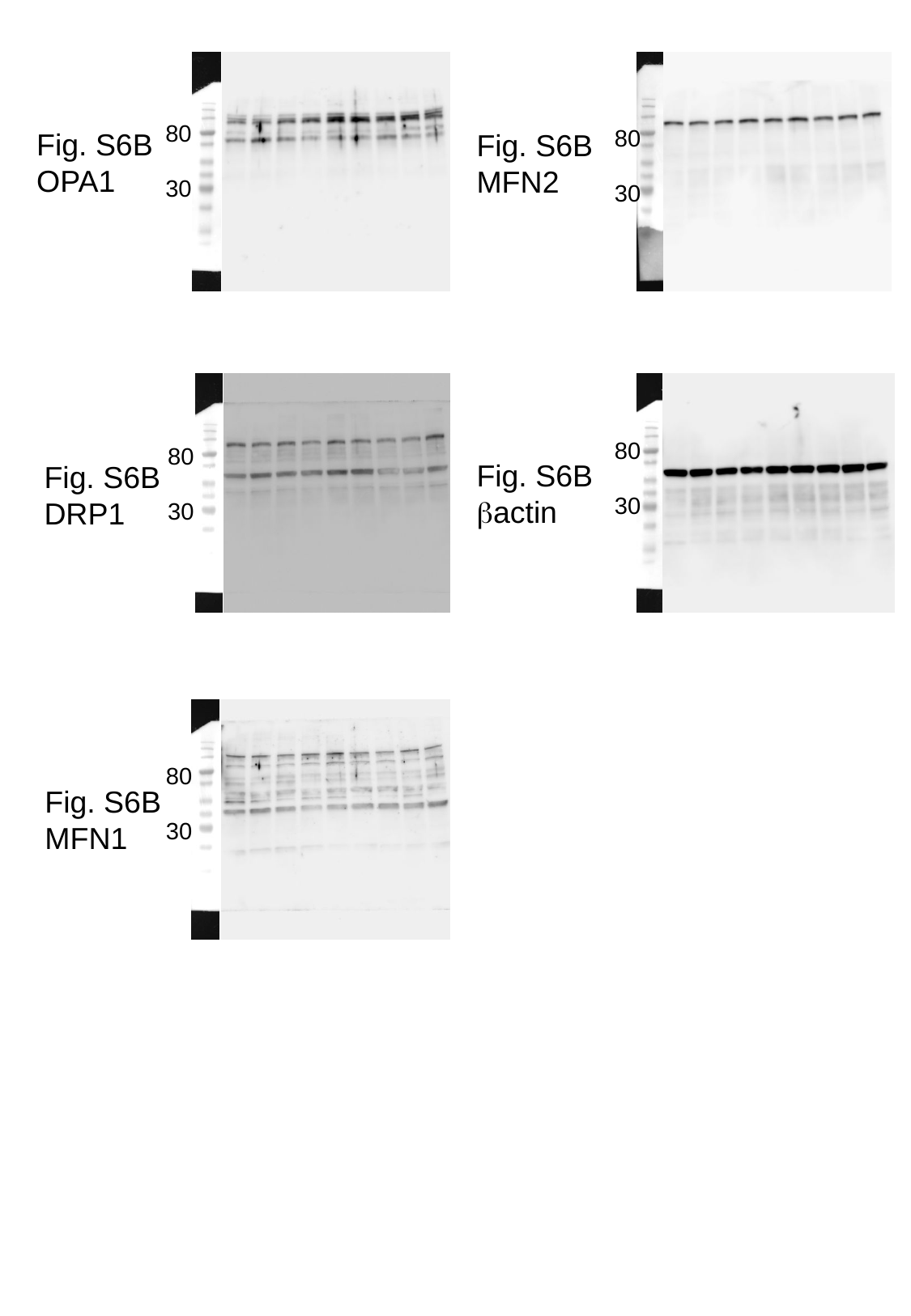

80
80
Fig. S6B
OPA1
Fig. S6B
MFN2
30
30
80
80
Fig. S6B
bactin
Fig. S6B
DRP1
30
30
80
Fig. S6B
MFN1
30

## Slide 3
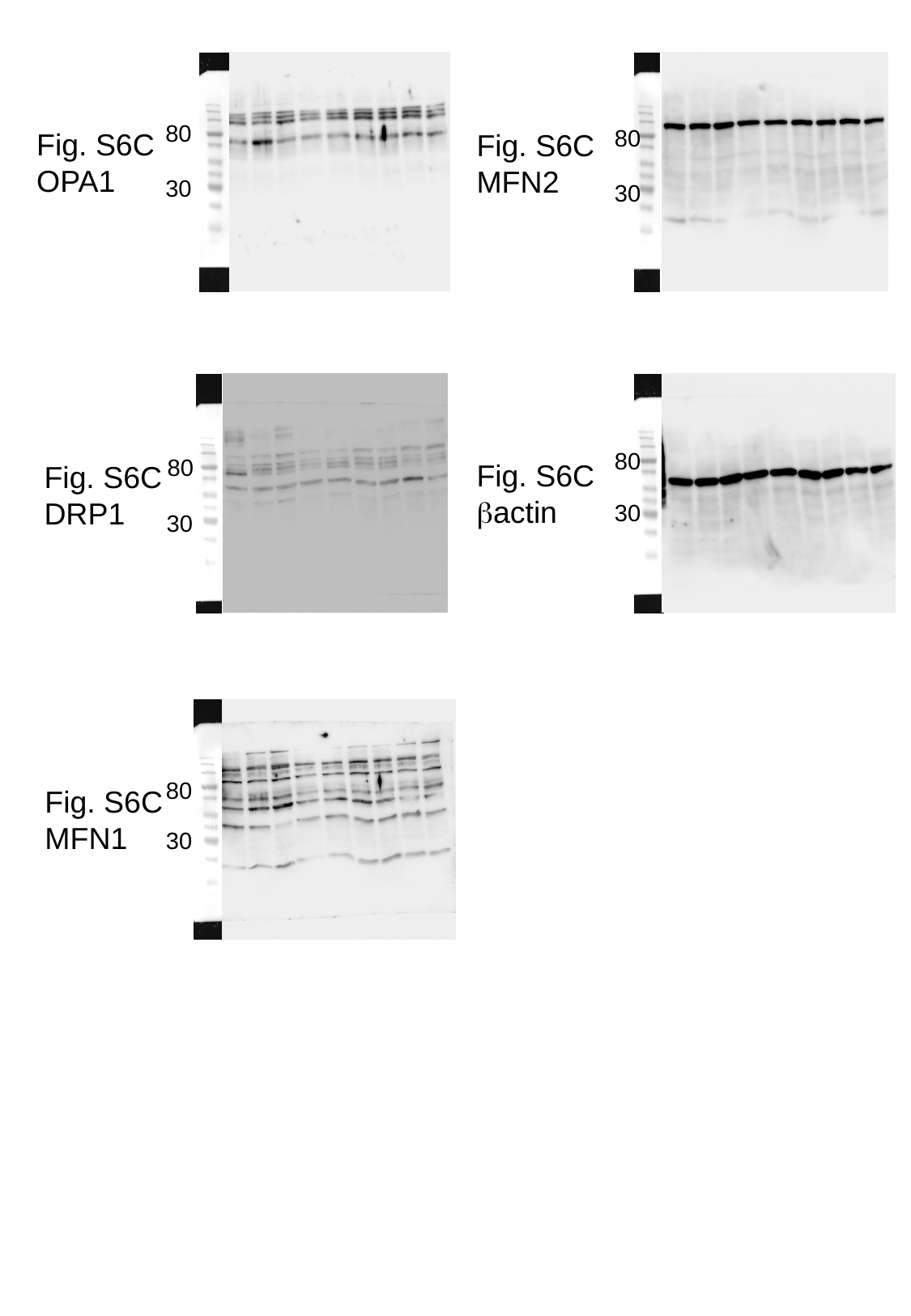

80
80
Fig. S6C
OPA1
Fig. S6C
MFN2
30
30
80
80
Fig. S6C
bactin
Fig. S6C
DRP1
30
30
80
Fig. S6C
MFN1
30

## Slide 4
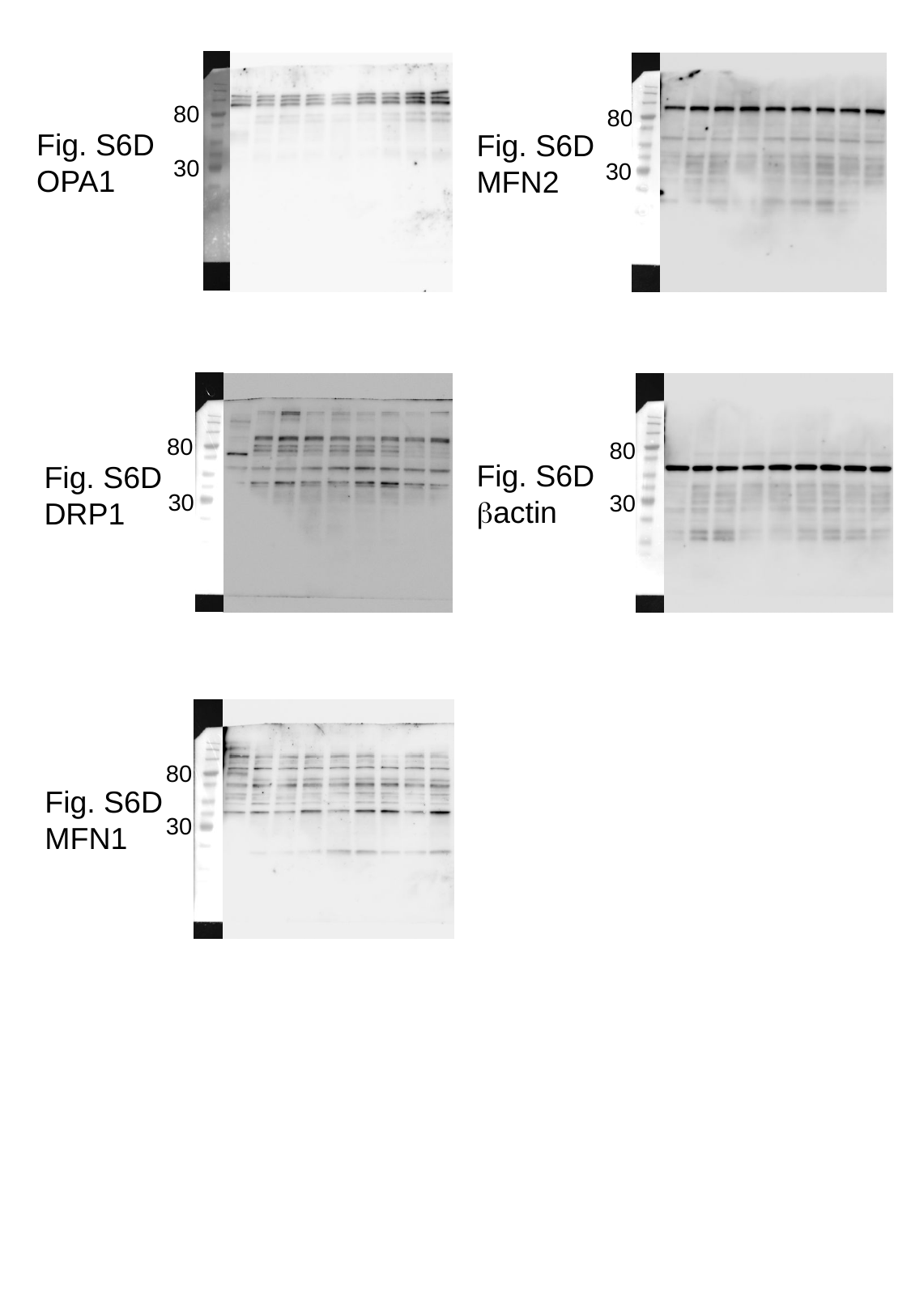

80
80
Fig. S6D
OPA1
Fig. S6D
MFN2
30
30
80
80
Fig. S6D
bactin
Fig. S6D
DRP1
30
30
80
Fig. S6D
MFN1
30
